# Interclass Switch between IL17 and IL23 Inhibitors in Psoriasis: A Real-Life, Long-Term, Single-Center Experience

**DOI:** 10.3390/jcm12247503

**Published:** 2023-12-05

**Authors:** Silvia Giordano, Paolo Dapavo, Michela Ortoncelli, Elena Stroppiana, Anna Verrone, Pietro Quaglino, Simone Ribero, Luca Mastorino

**Affiliations:** Dermatology Clinic, Medical Sciences Department, University of Turin, 10121 Torino, Italy; silvia.giordano94@gmail.com (S.G.); paolo.dapavo@gmail.com (P.D.); mortoncelli@cittadellasalute.to.it (M.O.); estroppiana@cittadellasalute.to.it (E.S.); averrone@cittadellasalute.to.it (A.V.); pietro.quaglino@unito.it (P.Q.); simone.ribero@unito.it (S.R.)

**Keywords:** psoriasis, IL23 inhibitors, IL17 inhibitors, PASI, switch, switching

## Abstract

Background: Interleukin 23 (IL-23) inhibitors, such as guselkumab, risankziumab, and tildrakizumab, have proved to be highly effective and safe for psoriasis treatment either in bio-naïve or bio-experienced patients. A substantial proportion of patients show a primary or secondary inefficacy to IL-17 inhibitors and can benefit from an alternative line of treatment, like IL-23 inhibitors. To date, no sufficient data are available on the effectiveness of IL-23 inhibitors after an anti-IL-17 agent. Methods: Our study includes 48 patients with moderate to severe psoriasis undergoing a switch from IL-17 to IL-23 inhibitors. This trial is registered with SS_DERMO_20. Results: The mean PASI (Psoriasis Area Severity Index) decreases from 11.6 to 3.3 at week 16, with responses maintained at weeks 28 and 52 (2 and 1.4, respectively), and a PASI100 achievement in more than 24% of patients at 16 weeks and 61.9 at 48 weeks, with no occurrence of serious adverse events. However, almost one in six patients interrupted the IL-23 inhibitors mainly due to primary ineffectivenss. Conclusions: Our data support the evidence that an interclass switch among IL-17 inhibitors is a safe and effective therapeutic option for these patients.

## 1. Introduction

Psoriasis, a chronic inflammatory skin disease, has been the focus of extensive research over the years [1]. Psoriasis affects approximately 3.2% of the adult population in the United States, and 0.13% of children. A total of 125 million people report having psoriasis, worldwide, with a prevalence of 0.5% in Asian countries and 8% in some European nations. Psoriasis can manifest at any age, without differences among sexes [2]. We can describe a bimodal age distribution for disease onset between 20–40 years old and between 50 and 70 years old [2,3]. Lesion traditionally affects the extensor areas of the limbs; it can also affect the folds, scalp, nails, genital region, palms, and soles. Lesions in plaque-type psoriasis manifest as erythematous, shiny, scaly, well-demarcated, and itchy plaques; in the case of fold involvement (the so-called inverse psoriasis), the lesions are smoother and less scaly [2,3].

Psoriasis can also affect hard-to-treat areas, such as the scalp, nails, and palmoplantar regions. The disease can also manifest as several clinical presentations, like erythrodermic psoriasis, characterized by a skin involvement superior to 90% of the body, and guttate psoriasis, appearing as tiny plaque and macules all over the body and often caused by an infection of the respiratory tract. A variant with a supposed different pathogenesis is pustular psoriasis, often associated with general involvement [2].

Psoriasis is frequently accompanied by several comorbidities. In particular, a correlation with metabolic syndrome is established, especially with the aspects of obesity and hypertension. Psoriasis is also associated with an increased risk of skin cancer. The impact on quality of life is high, sometimes compromising the usual tasks of affected patients [2,3].

Psoriasis treatment needs to be tailored according to the severity of the disease, and assessed with manageable scores.

The Psoriasis Area Severity Index (PASI) is a score ranging from 0 to 72 classically used for describing the severity and extension of the disease. The Physician Global Assessment (PGA-g), a 6-point score ranging from 0 (clear skin) to 5 (severe psoriasis), only provides information on the severity. Other scores, like the Psoriasis Scalp Severity Index and Palmoplantar Psoriasis Area Severity Index, are useful tools for disease assessment in the case of scalp and palmoplantar involvement [4].

A mild form of psoriasis can be managed by topical treatments, such as topical corticosteroids, vitamin D analogs, steroids plus vitamin D analog combinations, calcineurin inhibitors, keratolytics, and targeted phototherapy [2]. Phototherapy (narrow-band UVB, or PUVA) can be a suitable option for moderate forms of psoriasis, and traditional systemic treatments, such as methotrexate, cyclosporin, and acitretin, are still widely used for moderate-to-severe forms of psoriasis as first-line treatments [2].

Recent advancements have brought about a comprehensive revaluation of psoriasis’s underlying mechanisms, leading to the identification of novel molecular pathways. These newfound insights have paved the way for the development of targeted therapies that have proven to be remarkably efficacious in the management of this condition [5].

The emergence of biological agents in recent years has revolutionized psoriasis treatment. These agents, designed to selectively inhibit key molecules in the inflammatory cascade, have shown promising results. Notably, agents targeting tumor necrosis factor (TNF), IL-12/IL-23 p40 subunit, IL-17A, and IL-17RA have gained approval for treating psoriasis. This pivotal shift has significantly altered the therapeutic landscape, with particular emphasis on the superiority of anti-IL17 molecules, such as Ixekizumab and Secukinumab, over other biologics, including TNF inhibitors. This treatment also proved to be safe, without a significant role in tumor progression and latent tuberculosis infection reactivation [6,7]. However, it is important to note that, despite their efficacy, there is a potential for these anti-IL17 treatments to lose their effectiveness over time, necessitating the need to switch between biologics in clinical practice [1]. IL-17s can protect the host from extracellular pathogens, maintain epithelial integrity, regulate cognitive processes, and modulate adipocyte activity through distinct mechanisms. Its role in psoriasis pathogenesis was established by the 1990s [8].

In the last decade, the role of IL-23 has played a major role in psoriasis.

The main immunologic role of IL-23 is its involvement in the differentiation process of naive Th cells into Th17. Indeed, IL-23 indispensably contributes to the activation, maintenance, and proliferation after the initial induction driven by other cytokines, whose role is still debated. In mice, Th17 differentiation requires various cytokines, such as TGF-b and IL-6, which act coordinately with IL-21, an autocrine growth factor, and IL-23 [9]. Thus, the inhibition of this specific cytokine can allow a response also in patients where previous IL-17 inhibitors have failed. Since 2018, numerous IL-23 inhibitors have proved their efficacy and safety in the treatment of psoriasis and peripherical psoriatic arthritis, such as guslkumab, risankizumab, and tildrakizumab [10].

The concept of switching therapies for psoriasis management has garnered attention. Recently, Mastorino et al. reported switching to IL23 and IL-17 inhibitors after adalimumab failure. The authors showed no significant differences between adalimumab-experienced and bio-naive patients in the PASI100 and PASI90, and a PASI < 3 in patients treated with anti-IL17 agents. In patients treated with an anti-IL-23 agent, a faster response was observed in bio-naive patients, with the PASI < 3 score significantly higher than ADA-experienced patients at 16 weeks (77% vs. 58% *p* = 0.048). In a multivariate analysis of PASI100, only anti-IL-17 therapy appeared to have a negative impact at 52 weeks (OR: 0.54 *p* = 0.04) independently of a previous treatment [11].

Shifting from anti-IL17 to anti-IL-12/IL-23 and antiIL-23 treatments has been explored in previous studies, although clear correlations between their respective responses have yet to be established [12,13,14].

This retrospective analysis focuses on the experience of 48 patients from a tertiary center who underwent this specific switch in treatment.

## 2. Materials and Methods

### 2.1. Population

From our registry “Psocare SS-Dermo-20” at the Dermatology Unit of the University of Turin, we retrospectively analyzed the clinical data and medical history of patients with moderate to severe psoriasis from 1 January 2017 to 1 February 2023.

We collected the baseline characteristics, previous biological and non-biological therapies, and reasons for the switch. Regarding the latter, we considered primary failure as a non-achievement of the Psoriasis Area Severity Index, (PASI) < 5 from baseline at 6 months, while secondary failure was considered as a loss of PASI < 5 from baseline after 6 months, and side effects (other reasons). The data on effectiveness outcomes (Psoriasis Area Severity Index (PASI); PASI 100 and PASI 90) were collected until 52 weeks after the switch.

The study complied with the ethical standards of the 1975 Declaration of Helsinki.

The study was approved by our Internal Review Board (Comitato Etico Territoriale Interaziendale AOU Città della Salute e della Scienza di Torino) under the protocol SSDERMO20.

### 2.2. Objectives

The primary objective was to describe the effectiveness of interclass switching from IL-17 to IL-23 inhibitors.

The secondary objective was to describe if there could be significant baseline characteristics between the two populations: switched and non-switched patients.

### 2.3. Biological Therapies Considered

In the study, we considered the following treatment given at the traditional approved regimen after an initial specific induction (please refer to the specific datasheet):

- Ixekizumab (80 mg) fl sc every 4 weeks;
- Secukinumab (150 mg) 2 fl sc every 4 weeks;
- Brodalumab (210 mg) fl sc every 2 weeks;
- Guselkumab (100 mg) fl sc every 8 weeks;
- Risankizumab (75 mg) 2 fl sc every 12 weeks (150 mg fl was not available during the the study);
- Tildrakizumab (100 mg) fl sc every 12 weeks.

### 2.4. Statistical Analysis

Continuous variables were described by mean ± SD (standard deviation), based on the distribution of each variable. For the categorical variables, absolute and relative frequencies were provided. Percentages were based on the number of non-missing values. Inferential analyses were performed to compare the baseline characteristics (sex, age, age of onset, body mass index (BMI), joint involvement (PSA), cardiovascular comorbidities, diabetes mellitus, and obesity) of switched patients to the cohort of psoriatic patients treated with IL-17 inhibitors that did not switch to IL-23. The categorical variables were analyzed using chi-squared and Fisher’s exact tests where appropriate, while the continuous variables were tested using the Shapiro–Wilk test to investigate the normality of the distribution. Dichotomous normal distributions were compared using the student’s *t*-test, and non-normal distributions were tested using the Mann–Whitney U test.

A statistical analysis was conducted using STATA 15.1 SE (StataCorp., College Station, TX, USA, 2017); all the tests were two-sided and the statistical significance was set to *α* = 0.05.

## 3. Results

Within the reported timeframe, out of 1057 patients with moderate to severe psoriasis under the care of the Dermatology Clinic at Turin University Hospital, 638 received anti-IL17 drugs. Among them, a subset of 48 patients (7.5%) transitioned from anti-IL17 therapy to an IL-23 inhibitor due to treatment failure.

The patient demographic was slightly skewed towards females, constituting 36.7% of the population, with a mean age of 56.5 years old (SD 13). The onset of psoriasis occurred at a mean age of 35.7 years old (SD 16.5), and the average duration of diagnosis spanned 252 months. The cohort exhibited a mean BMI of 27.2 kg/m^2^ (SD 5.3), and notably, 18.8% were classified as obese. Furthermore, 11.4% of patients presented with type-two diabetes, while a significant proportion, 65.7%, had concurrent cardiovascular conditions, encompassing arterial hypertension, atherosclerotic disease, and coronary artery disease. In contrast to the 590 non-switched patients, there were no discernible differences in the baseline characteristics, except for cardiovascular comorbidity, which was notably higher in the switched group (65.7% vs. 44%, *p* = 0.024) (refer to Table 1 and Supplementary Materials). Interestingly, more than half of these patients (58.3%) experienced the switch as a primary treatment failure, while for 39.6%, it marked the second instance of treatment failure. Only one patient switched due to adverse events.

Exploring their treatment histories revealed a diverse landscape of non-biologic therapies before the adoption of biologics. Phototherapy was employed in a modest fraction of the cases, accounting for 10.4%, while the majority underwent systemic therapy. Among these, methotrexate featured prominently, utilized in 50% of the cases, followed by acitretin (33.3%) and cyclosporin (37.5%). Intriguingly, 31.3% of the patients had not undergone any non-biologic drug treatments. The transition from anti-IL17 to anti-IL-23 therapies was characterized by a direct shift, devoid of wash-out periods or intervening treatments. Among the 48 patients who made the switch, 14 opted for guselkumab (29.2%), 31 for risankizumab (64.6%), and 3 for tildrakizumab (6.2%). A substantial majority (83.3%) experienced a seamless transition from anti-IL17 to anti-IL-23 therapies, while a smaller subset (16.7%) had previously undergone an intraclass switch. During the mean follow-up time of 11.4 months (SD 5.2) for the anti-IL-23 treatment, four patients discontinued Risankizumab and three discontinued Guselkumab, constituting a discontinuation rate of 14.3%. The ineffectiveness of the treatment emerged as the primary reason for the discontinuation. Notably, the patients reported no adverse events during the follow-up period, as detailed in Table 1.

**Figure 1:**
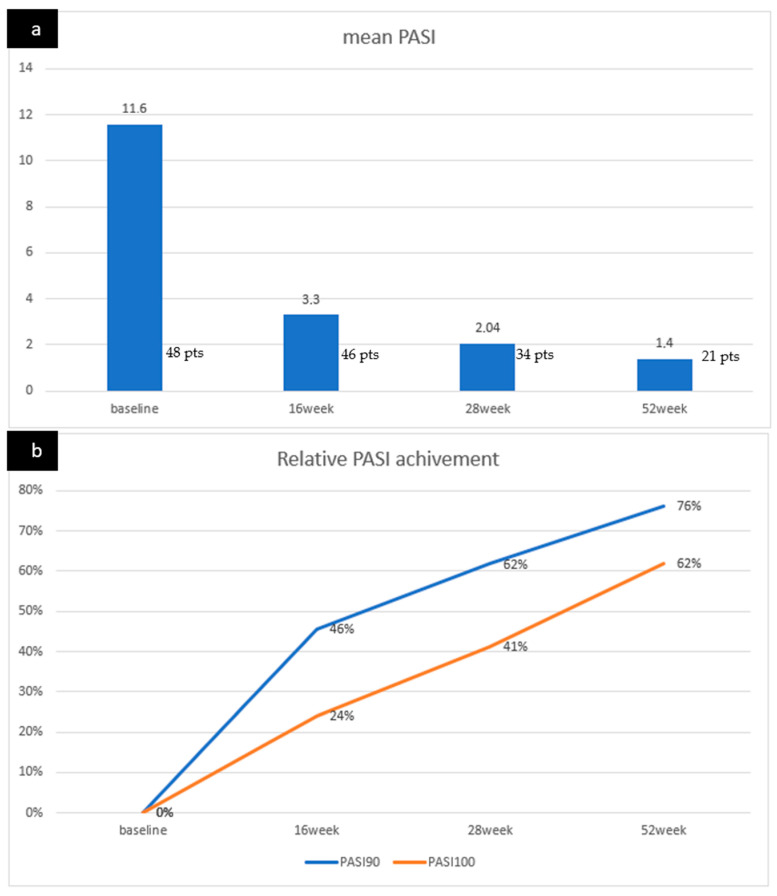
(**a**) Mean PASI scores at baseline, 16, 28, and 52 weeks. (**b**) PASI 100 and 90 scores increase until 48 weeks for 61.9% and 76.2% of patients, respectively.

In the context of disease activity, the initial mean PASI score stood at 11.6 (SD 5.8) during the transition, showing a substantial decrease to 3.3 (SD 3.5) after the initial 16 weeks. Noteworthy is the fact that, at the 16-week mark, 11 patients exhibited a remarkable achievement of PASI 100, constituting 24% of the cohort, while 21 patients achieved PASI 90, accounting for 45.7% of the participants. The positive response observed during this period persisted in the subsequent weeks, reflecting mean PASI scores of 2 (SD 2.5) at week 28 and 1.4 (SD 2.3) at week 52. The trend of success in reaching PASI scores of 100 and 90 continued to ascend, with 61.9% of patients achieving PASI 100 and 76.2% achieving PASI 90 by week 48 (Figure 1).

## 4. Discussion

While the concept of transitioning from anti-IL17 to anti-IL-23 therapies is relatively novel, the emerging evidence from this study and others provide compelling support for its safety and efficacy. The switch led to rapid and progressive clinical improvements in psoriasis management. The mechanism underlying this phenomenon remains partially understood, but it has been theorized that IL-23’s role in both promoting IL-17 pathways and enhancing anti-inflammatory T-regulatory cells contributes to the positive outcomes observed [12,15,16]. Few experiences allow a similar consideration for another inflammatory disease, like hidradenitis suppurativa [17].

The well-established efficacy of anti-IL-23, as indicated by numerous real-life studies, including recent ones [18], has demonstrated its consistent maintenance even after the prior use of anti-IL17 inhibitors; yet, there is a notable scarcity of research dedicated to evaluating its effectiveness, specifically after treatment switching [19,20,21]. A recent study showed a longer drug survival rate in favor of IL-23 inhibitors [14]. Bonifati et al., in a small case series, reported the efficacy of switching to guslkumab and risankizumab after therapeutic failure with ixekizumab and secukinumab confirming our results [22]. These authors showed in a pre-post analysis a significant reduction in the mean PASI, DLQI, VASitch, and PGA, already at 3 months, with a maintained response at 6 months [22].

Megna and colleagues reported on the effectiveness of risankizumab after the therapeutic failure of IL-17 inhibitors, also evaluating hard-to-treat localization [23].

In this study, a total of eight patients were enrolled. A total of 62.5% of the patients received ustekinumab, seven (87.5%) at least one anti-IL17 treatment, and only one (12.5%) patient received guselkumab. Secukinumab was used in five (62.5%) cases, and ixekizumab in four (50.0%). The authors showed a reduction in the mean PASI and BSA scores from 11.9 ± 5.5 and 22.9 ± 13.1 and 3.3 ± 1.7 and 7.5 ± 5 (*p* < 0.001 and *p* < 0.01) at week 16, respectively. The mean baseline NAPSI (18.0 ± 8.5) reduced to 7 ± 1.4 at week 16. Palmo-plantar and scalp areas showed reductions of 67.5% and 99.9% at week 16, respectively [23].

Hung and colleagues highlighted that previous exposure to IL-17 inhibitors was significantly associated with reduced PASI 75 responses at weeks 12, 20, and 28 (OR = 0.19, 0.10, and 0.03, respectively). On the contrary, no association was observed in patients previously receiving TNF-α or IL-12/23 inhibitors [24].

Chiang et al. suggested that switching from IL-17 to IL-23 inhibitors could also be a feasible option for erythrodermic psoriasis [25].

There are examples of the ‘reverse-switch strategy’ in the literature. Mastorino et al. reported that seven patients switched to an IL17 inhibitor after treatment failure with an IL23 inhibitor. Four patients switched to brodalumab and three to ixekizumab; one patient performed a rechallenge with ixekizumab. The initial mean PASI at the switch was 8.3 (ds 2.3) and after 16 weeks dropped to 1.1 (ds 2.0), with five out of seven patients achieving PASI100; in the following weeks, the response was maintained with mean PASI scores of 1.4, 1.4, and 1.6 at weeks 24, 40, and 52, respectively [16].

Damiani et al. reported a successful switch from secukinumab to adalimumab or ustekinumab in 50 psoriatic patients. Secukinumab was discontinued for a lack of efficacy at 16 weeks in 15 cases, for loss of efficacy in 20 cases, for infectious reasons (fungal/bacterial infections) in 7 cases, and for other causes in 8 cases. A total of 29 patients who switched to adalimumab showed a mean PASI of 2.8 ± 0.9 and DLQI 6 ± 2.3 at week 52, if they were biologically naive patients prior to secukinumab administration, and 3.1 ± 0.4 and 8 ± 1.2 if they received ustekinumab prior to starting secukinumab use. A total of 21 patients switched to ustekinumab. At week 52, the PASI score was 2.6 ± 0.3 and DLQI 4.1 ± 3.7 for biologically naive patients prior to secukinumab use, and 3.8 ± 0.2 and 10 ± 2.1 for those who received TNFi prior to starting secukinumab [13].

Similarly, Chiricozzi et al. reported on 21 patients who switched from secukinumab to ustekinumab. Ustekinumab was effective in reducing disease severity, with significant improvements in both psoriasis area severity index and dermatology quality of life index (DLQI) scores. PASI score improvements of 31.8, 44, 77.8, 80.3, 80.5, and 89.6% at weeks 4, 12, 24, 36, 48, and over 60 weeks, respectively, were detected (*p* < 0.05), achieving PASI responses of 50, 75, and >90 for 93.8, 87.5, and 50% of patients at week 48 [12].

In our population, a mean reduction in the PASI after a switch > 5 points was observed, with PASI 100 being achieved by more than 20% of patients after only 16 weeks. A significant number of patients interrupted their treatment with guselkumab and risankizumab, with almost 1/6 failing, while no cases of failure with tildrakizumab were recorded, considering the low number of patients on this drug. However, regarding safety, none of our 48 patients had to discontinue the treatment because of adverse events. In general, the curves reveal a progressive, but slower, response than that reported in the registration trials. Although IL-23 inhibitors are generally effective after IL-17 inhibitor failure, they are generally more effective in bio-naive patients, highlighting the importance of appropriate treatment selection based on patient characteristics [20,21]. The BADBIR study by Yu et al. showed that, with each line of therapy, treatment survival was reduced; interestingly, guslkumab, in this real-life experience, was the most effective, even when used as a subsequent line of therapy [26]. The reduced treatment survival and the switch result had a high economic impact by delaying the achievement of the ideal therapeutic targets for the psoriatic patient [27]. Despite the confirmed effectiveness of the switching strategy after previous IL-inhibitors or anti-TNFalfa therapies, this strategy appeared to be the most effective out of the proper targets used for psoriasis management [11,27]. An idealistic therapeutic strategy aims to achieve PASI 90 and DLQI 0/1 scores in the first 16 weeks of treatment, maintaining a long drug survival rate and avoiding the use of the switching strategy and side effects. To conclude, a therapeutical switch is an option, but it is also a mark of incorrect patient selection [27].

The limitations of the present study reside in the inherent limitations of a real-life-experience study (i.e., retrospective nature and observed cases analysis) and in the small cohort evaluated. Consequently, our inability to identify the potential correlations with baseline population characteristics and any potential prognostic factors underscores the necessity for further investigations to definitively establish this link.

## 5. Conclusions

In conclusion, this retrospective analysis sheds light on the experiences of patients who switch from anti-IL17 to anti-IL-23 therapies for psoriasis. The findings highlight the potential benefits of this transition after treatment failure and emphasize the viability of anti-IL-23 agents as effective alternative treatment. While further research using larger cohorts is necessary to validate these findings, this study provides valuable insights for clinicians navigating the complexities of psoriasis treatment.

## Figures and Tables

**Table 1:** Baseline patients characteristics.

	Switched Patients (48)	General Psoriatic Population (590)	*p*-Value
Sex (F/M)	36.7%	35.7%	0.659
Age (mean)	56.5 (SD 13)	54.7 (SD 15.6)	0.429
Age of onset (mean)	35.7 (SD 16.5)	35.65 (16 SD)	0.819
PSA (yes/no)	16.7%	26.9%	0.117
BMI (mean)	27.2 (SD 5.3)	27 (SD 5.6)	0.225
Obesity (yes/no)	18.8%	18.8%	0.985
Cardiovascular disease (yes/no)	65.7%	44%	0.024
Diabetes mellitus (yes/no)	11.4%	12.7%	0.688

## Data Availability

Data available upon request.

## Supplementary Materials

The following supporting information can be downloaded at: https://www.mdpi.com/article/10.3390/jcm12247503/s1, Table S1. General characteristics of the 48 patients that switched from IL17 to Il23 inhibitors and the PASI score trend from baseline to 52 weeks.
