# Supplementary material for: Interclass Switch between IL17 and IL23 Inhibitors in Psoriasis: A Real-Life, Long-Term, Single-Center Experience
Source: J Clin Med. 2023 Dec 5;12(24):7503. doi: 10.3390/jcm12247503 (PMC10743732; doi:10.3390/jcm12247503)
Supplement: Supplementary file 1 [file jcm-12-07503-s001.zip › jcm-2679283-supplementary.pdf]

**Table S1.** General characteristics of the 48 patients that switched from IL17 to Il23 inhibitors and the PASI score trend from baseline to 52 weeks.

| No. of patients | Age | Sex | BMI | Age of onset | Type of psoriasis | PSA | Biologicals in course | Previous biological therapy | PASI baseline | PA SI 16weeks | PASI 90 16weeks | PASI 1 00 16weeks | PASI 28 weeks | PASI 90 28weeks | PA SI 100 28weeks | PASI 48weeks | PAS I00 48weeks | PA SI 100 48weeks | Follow up (months) | Cause of IL17 inhibitors suspension | Obesity | Cardiovascular disease | Diabetes mellitus |
|---|---|---|---|---|---|---|---|---|---|---|---|---|---|---|---|---|---|---|---|---|---|---|---|
| 1 | 62 | m | 27 | 47 | vulgar | no | risankizumab | adalimumab ixekizumab | 9 | 3 | no | no | NA | NA | NA | NA | NA | NA | 8 | | no | yes | no |
| 2 | 61 | m | 28 | 20 | vulgar | yes | risankizumab | adalimumab, certolizumab, secukinumab | 6 | 6 | no | no | NA | NA | NA | NA | NA | NA | 3 | primary inefficacy | no | no | no |
| 3 | 65 | f | 26 | 24 | vulgar | yes | risankizumab | adalimumab, etanercept, certolizumab, ixekizumab | 15 | 0 | yes | yes | 0 | yes | yes | NA | NA | NA | 9 | | no | yes | no |
| 4 | 67 | f | 27 | 47 | vulgar | no | risankizumab | adalimumab, etanercept, infliximab, ixekizumab | 11 | 0 | yes | yes | 0 | yes | yes | 0 | yes | yes | 14 | | no | no | no |
| 5 | 78 | f | 35 | 23 | vulgar | no | risankizumab | adalimumab, etanercept, ixekiumab | 16 | 5 | no | no | NA | NA | NA | NA | NA | NA | 6 | | yes | yes | no |
| 6 | 60 | m | 30 | 23 | vulgar | no | guselkumab | adalimumab, etanercept, secukinumab | 10 | NA | NA | NA | 4 | no | no | 3 | no | no | 18 | | yes | NA | NA |
| 7 | 67 | f | 43 | 27 | vulgar | no | risankizumab | adalimumab, infliximab, ixekizumab, brodalumab | 19 | 1.5 | yes | no | 1.5 | yes | no | NA | NA | NA | 10 | | yes | yes | yes |
| 8 | 57 | f | 26 | 47 | vulgar | yes | tildrakizumab | adalimumab, ixekizumab | 11 | 0 | yes | yes | 0 | yes | yes | 0 | yes | yes | NA | | no | NA | NA |
| 9 | 65 | m | 29 | 41 | vulgar | no | risankizumab | adalimumab, ixekizumab, brodalumab | 17 | 1.5 | yes | no | 0 | yes | yes | NA | NA | NA | 11 | | no | yes | yes |
| 10 | 53 | m | 23 | 44 | vulgar | no | guselkumab | adalimumab, secukinumab | 12 | 3 | no | no | 1 | yes | no | 1 | yes | no | 15 | | no | NA | NA |
| 11 | 58 | m | 25 | 49 | vulgar | no | risankizumab | adalimumab, secukinumab | 8 | 4 | no | no | 0 | yes | yes | NA | NA | NA | 10 | | no | yes | no |
| 12 | 54 | m | 26 | 37 | vulgar | no | risankizumab | adalimumab, secukinumab, ixekizumab, brodalumab | 8 | 3 | no | no | 1.5 | yes | no | NA | NA | NA | 11 | | no | yes | no |
| 13 | 42 | m | 24 | 15 | vulgar | yes | guselkumab | adalimumab, ustekinumab, ixekizumab | 10.8 | 6 | no | no | 5 | no | no | 0 | yes | yes | 21 | | no | NA | NA |
| 14 | 58 | m | 37 | 38 | vulgar | yes | guselkumab | adalimumab, ustekinumab, secukinumab | 22 | 2 | yes | no | 0.5 | yes | no | 0 | yes | yes | 21 | | yes | yes | yes |

| | | | | | | | | | | | | | | | | | | | | | | | |
|---|---|---|---|---|---|---|---|---|---|---|---|---|---|---|---|---|---|---|---|---|---|---|---|
| 15 | 41 | f | 22 | 26 | vulgar | no | risankizumab | adalimumab, ustekinumab, secukinumab | 7 | 2 | no | no | NA | NA | NA | NA | NA | NA | 8 | | no | NA | NA |
| 16 | 65 | f | 26 | 22 | erythrodermic | no | guselkumab | adalimumab,etanercept,inflximab, ustekinumab, ixekizumab | 30 | 16. | no | no | 8 | no | no | 7 | yes | no | 14 | lack of efficacy | no | NA | NA |
| 17 | 42 | m | 28 | 34 | vulgar | no | risankizumab | brodalumab | 8 | 0 | yes | yes | 0 | yes | yes | 0 | yes | yes | 13 | | no | no | no |
| 18 | 72 | f | 22 | 54 | vulgar | no | risankizumab | etanercept secukinumab | 6 | 3 | no | no | 3 | no | no | 5 | no | no | 13 | | no | no | no |
| 19 | 58 | m | 23 | 36 | vulgar | yes | guselkumab | etanercept, infliximab, ixekizumab | 5 | 3 | no | no | 3 | no | No | 3 | no | no | 23 | | no | yes | no |
| 20 | 53 | m | 27 | 23 | vulgar | no | risankizumab | etanercept, ixekizumab | 10 | 4 | no | no | 1 | yes | No | 1 | yes | no | 14 | | no | yes | no |
| 21 | 51 | m | 23 | 26 | vulgar | no | risankizumab | infliximab, ustekinumab, secukinumab | 8 | 1.5 | yes | no | 1.5 | yes | No | NA | NA | NA | 9 | | no | yes | no |
| 22 | 46 | m | 35 | 34 | vulgar | no | risankizumab | ixekizumab | 0 | 0 | yes | yes | NA | NA | NA | NA | NA | NA | 8 | | yes | yes | no |
| 23 | 70 | f | 19 | 45 | vulgar | no | risankizumab | ixekizumab | 9 | 2 | no | no | NA | NA | NA | NA | NA | NA | 8 | | no | yes | no |
| 24 | 65 | m | 25 | 53 | vulgar | no | risankizuma | ixekizumab | 10 | 1.5 | no | no | NA | NA | NA | NA | NA | NA | 8 | | yes | yes | no |
| 25 | 74 | f | 24 | 74 | vulgar | no | guselkumab | ixekizumab | 12 | 0 | yes | yes | 0 | yes | yes | 0 | yes | yes | 22 | | no | NA | NA |
| 26 | 62 | m | 27 | 46 | vulgar | no | risankizumab | ixekizumab | 8 | 15 | no | no | NA | NA | NA | NA | NA | NA | 5 | primary inefficacy | no | yes | no |
| 27 | 73 | m | 23 | 50 | vulgar | no | risankizumab | ixekizumab | 17 | 1.5 | yes | no | NA | NA | NA | NA | NA | NA | 9 | | no | yes | no |
| 28 | 65 | f | 25 | 4 | vulgar | yes | guselkumab | ixekizumab | 7 | 8 | no | no | NA | NA | NA | NA | NA | NA | 4 | primary inefficacy | no | no | no |
| 29 | 46 | m | 28 | 35 | vulgar | no | guselkumab | ixekizumab | 11 | 0 | yes | yes | 0 | yes | yes | 0 | yes | yes | 17 | | no | NA | NA |
| 30 | 35 | m | 20 | 9 | vulgar | no | guselkumab | ixekizumab | 12 | 0.5 | yes | no | 0 | yes | yes | 0 | yes | yes | 18 | | no | NA | NA |
| 31 | 42 | m | 35 | 3 | guttate | no | risankizumab | ixekizumab | 10 | 3 | no | no | NA | NA | NA | NA | NA | NA | 7 | | no | no | no |
| 32 | 43 | f | 25 | 41 | vulgar | yes | risankizumab | ixekizumab, brodalumab | 16 | 10 | no | no | 7 | no | no | NA | NA | NA | 8 | primary inefficacy | no | yes | no |

| | | | | | | | | | | | | | | | | | | | | | | | |
|---|---|---|---|---|---|---|---|---|---|---|---|---|---|---|---|---|---|---|---|---|---|---|---|
| 33 | 67 | m | 27 | 45 | vulgar | no | risankizumab | ixekizumab, brodalumab | 30 | 12 | no | no | 8 | no | no | NA | NA | NA | 10 | | no | yes | no |
| 34 | 75 | m | 41 | 50 | vulgar | no | risankizumab | ixekizumab, brodalumab | 18 | NA | NA | NA | NA | NA | N A | N A | NA | NA | 1 | | yes | yes | no |
| 35 | 67 | f | 28 | 60 | inverse | no | tildrakizumab | secukinumab | 7 | 0 | yes | yes | 0 | yes | yes | 0 | yes | yes | 9 | | no | yes | no |
| 36 | 71 | f | 20 | 54 | vulgar | no | tildrakizumab | secukinumab | 17 | 1.5 | yes | no | 0 | yes | yes | 0 | yes | yes | 9 | | no | | |
| 37 | 77 | m | 30 | 68 | vulgar | no | risankizumab | secukinumab | 8 | 1.5 | no | no | 1.5 | no | no | 0 | yes | yes | 12 | | no | yes | yes |
| 38 | 53 | m | 23 | 21 | vulgar | no | guselkumab | secukinumab | 13 | 6.5 | no | no | 3.5 | no | no | 7 | no | no | 22 | | no | NA | NA |
| 39 | 59 | m | 36 | 54 | vulgar | no | risankizumab | secukinumab | 8 | 6 | no | no | 6 | no | no | NA | NA | NA | 8 | primary inefficacy | yes | no | no |
| 40 | 55 | m | 27 | 36 | vulgar | no | risankizumab | secukinumab | 10 | 6 | no | no | 3 | no | no | NA | NA | NA | 11 | | no | yes | no |
| 41 | 44 | f | 24 | 38 | vulgar | no | risankizumab | secukinumab | 6 | 0 | yes | yes | 0 | yes | ye s | NA | NA | NA | 11 | | no | yes | no |
| 42 | 32 | m | 19 | 17 | vulgar | no | guselkumab | secukinumab | 5.5 | 0 | yes | yes | 0 | yes | ye | 0 | yes | yes | 16 | | no | | |
| 43 | 22 | f | 23 | 8 | vulgar | no | risankizumab | secukinumab brodalumab | 10 | 3 | no | no | NA | NA | NA | NA | NA | NA | 7 | | no | no | no |
| 44 | 27 | m | 30 | 12 | vulgar | no | risankizumab | secukinumab ustekinumab | 15 | 0 | yes | yes | 0 | yes | yes | NA | NA | NA | 13 | | no | no | no |
| 45 | 50 | m | 28 | 26 | vulgar | no | guselkumab | secukinumab, ixekizumab | 6 | 1.5 | yes | no | 6 | no | no | NA | NA | NA | 7 | primary inefficacy | no | NA | NA |
| 46 | 48 | m | 26 | 28 | vulgar | no | risankizumab | ustekinumab secukinumab | 9 | 1.5 | yes | no | NA | NA | NA | NA | NA | NA | 7 | | no | no | no |
| 47 | 53 | f | 26 | 49 | guttate | no | risankizumab | ustekinumab, ixekizumab | 17 | 1.5 | yes | no | 1.5 | yes | no | 0 | yes | yes | 13 | | no | no | no |
| 48 | 64 | m | 34 | 50 | vulgar | no | guselkumab | ustekinumab, secukinumab | 17 | 5 | no | no | 3 | no | no | 3 | no | No | 16 | | yes | NA | NA |
